# Cancer risk in tuberculosis patients in a high endemic area

**DOI:** 10.1186/s12885-021-08391-6

**Published:** 2021-06-09

**Authors:** Guang-Liang Chen, Li Guo, Shun’e Yang, Dong-Mei Ji

**Affiliations:** 1 Department of Medical Oncology, Fudan University Shanghai Cancer Center, No. 270, Dong’an Road, Xuhui District, Shanghai, 200032 China; 2 Department of Oncology, Shanghai Medical College Fudan University, No. 270, Dong’an Road, Xuhui District, Shanghai, 200032 China; 3 Department of Oncology, Xinjiang Cancer Hospital, Xinjiang Medical University, Xinjiang, 830000 China; 4 Phase I Clinical Trial Center, Fudan University Shanghai Cancer Center, No. 4333, Kangxin Road, Pudong New District, Shanghai, 201135 China

**Keywords:** Tuberculosis, Malignant Cancer, Case-control study

## Abstract

**Background:**

Tuberculosis (TB) may facilitate carcinogenesis. We performed a case-control study of the association between TB and cancer in Xinjiang, a high TB endemic area of China.

**Methods:**

From January 2016 to December 2018, a total of 45,455 patients hospitalized in Xinjiang Cancer Hospital were consecutively enrolled and divided into a malignant tumor group (*n* = 32,539) and a benign tumor group (*n* = 12,916). Patients with active and previous TB before the diagnosis of cancer were retrospectively identified in the two groups.

**Results:**

A significantly higher proportion of TB was found in the malignant tumor group (*n* = 1776, 5.46%) than in the control (benign tumor) group (*n* = 175, 1.35%) (*p* < 0.0001). The highest and lowest proportions of TB in the malignant group were in patients with non-Hodgkin’s lymphoma (16.74%) and thyroid cancer (0.77%), respectively. In multivariate analysis adjusting for age, sex, and ethnicity, TB remained an independent risk factor for all cancers (odds ratio (OR) 1.68; 95% confidence interval (CI) 1.43–1.97). Furthermore, TB was associated with a significantly higher risk of non-Hodgkin’s lymphoma, cervical cancer, esophageal cancer, “other” cancers, ovarian cancer, and breast cancer. Moreover, females with TB were more likely to develop cancer than males (*p* < 0.0001), except for esophageal cancer and lymphoma.

**Conclusion:**

TB patients have an elevated cancer risk. A screening strategy for TB should be taken into consideration before treatment in patients with some cancer types that are associated with a high proportion of TB.

## Background

Tuberculosis (TB) is an old disease that has affected humans more than 8000 years ago [1, 2]. To date, most TB is completely curable with a timely diagnosis and correct drug treatment [2, 3]. Globally, in 2015, the age-standardized tuberculosis incidence rates (per 100 000 people) among men and women were 154.4 (140.0–172.2) and 86.3 (78.0–97.4), respectively [4]. The age-standardized tuberculosis mortality rates (per 100 000 people) among men and women were 21.9 (16.5–;29.5) and 10.8 (8.5–13.1), respectively [4]. With declining trends in mortality, incidence, and prevalence of TB in human immunodeficiency virus (HIV)-negative individuals, challenges remain in most Asia [4, 5].

Western China is a high endemic area for TB [6, 7]. The patient diagnosis rate of pulmonary TB was 0.34 (95% confidence interval (CI) 0.25–0.44) in the 2010–2011 Xinjiang survey [6]. In addition, the burden of multidrug-resistant and extensively drug-resistant TB cases was also found to be substantial in the Xinjiang area [7]. TB may induce a chronic inflammatory state and impair T-cell-mediated immunity, contributing to the development of cancer [8–10]. Indeed, a number of epidemiologic studies have shown that the risk of cancer is the greatest in the first 2 years after the diagnosis of TB [9–13] but remains elevated for long periods [12, 14-18]. Understanding the risk of the development of cancer after TB in a highly endemic area has some practical clinical significance, such as improving the prevention and diagnosis of cancer.

However, limited data are available from Western China to support the link between cancer and TB. Data from such areas might add valuable insights into the relationship between TB and malignant cancer. Therefore, we performed a retrospective case-control study to compare the proportion of TB in malignant tumor patients and benign tumor patients.

## Methods

### Patient selection method

From January 2016 to December 2018, a total of 46,371 inpatients were treated at Xinjiang Cancer Hospital, a tertiary cancer center in Xinjiang. This cohort was then divided into two groups, namely, the malignant tumor group (*n* = 32,539) and the control (benign tumor) group (*n* = 12,916) (Table 1), based on the definitive pathological diagnosis confirmed by at least 2 experienced pathologists. Patients who were diagnosed with TB after being diagnosed with cancer (*n* = 272) and those lacking definitive pathological reports (*n* = 644) were excluded from this study. This study was performed in accordance with the principles of the Declaration of Helsinki.

**Table 1:** Cancer risk in patients with tuberculosis (TB) stratified by patient characteristics

Characteristics	Malignant tumor group (***N*** = 32,539)		Benign tumor group (***N*** = 12,916)		***P***
	No	%	No	%	
Sex					< 0.0001
Male	12,420	38.17	791	6.12	
Female	20,119	61.83	12,125	93.88	
Age, years					< 0.0001
Median	55.0	–	41.4	–	
Range	2–99	–	5–91	–	
Ethnicity					0.2155
Han	21,845	67.13	8734	67.62	
Non-Han	10,694	32.87	4182	32.38	
**Tuberculosis**					< 0.0001
TB	1776	5.46	175	1.35	
Non-TB	30,763	94.54	12,741	98.65	

### Identification of tuberculosis patients

A retrospective TB survey was performed in the two groups. Patients with active and previous TB before the diagnosis of cancer (benign or malignant) were obtained from the Department of TB Registry in Xinjiang Cancer Hospital. Among the TB patients, 1673 had pulmonary TB, 103 had extrapulmonary TB in the malignant tumor group, and 153 and 22 patients had pulmonary and extrapulmonary TB in the control group, respectively. In the malignant tumor group, most patients with TB disease had previous pulmonary TB, according to clinical and radiological indicators such as old pulmonary TB (*n* = 1690). Active pulmonary TB was found in 78 patients in the malignant tumor group who were positive for smear or culture results (*n* = 12) or had radiographic abnormalities consistent with active pulmonary TB (*n* = 66). In the control group, 167 patients had previous pulmonary TB, and only 2 patients had radiographic abnormalities consistent with active pulmonary TB. Only 8 and 6 patients in the malignant tumor and control groups, respectively, had a past medical history of pulmonary TB diagnosed by their doctors.

In both groups, when patients were hospitalized multiple times, only the first hospitalization was included in this study. “Other cancer” refers to any cancer defined by type or site accounting for less than 5%, including cancer originating in the testis, vulva, penis, vagina, labia majora, labia minora, perineum, scrotum, hydatidiform mole, chorion, notochord, adrenal cortex, neuroendocrine system, mediastinum, sweat glands, mesothelium, thymus, or umbilical tube.

### Statistical analysis

Data were analyzed using SPSS (version 25.0; SPSS, Chicago, IL). To identify the risk of cancer associated with TB, age, sex, and ethnicity were adjusted for in the statistical analysis. A binary logistic regression model was used to estimate the adjusted odds ratio (OR) and 95% confidence interval (CI). The variables related to the event of interest (cancer) were introduced into the model in an unconditional way. A 2-tailed *P* value < 0.05 was considered statistically significant.

## Results

### TB occurred more frequently in malignant cancer than in benign tumor patients

To assess the relationship between TB and tumors, we assigned the inpatients to two groups: the control group (benign tumor group, *n* = 12,916) and the study group (malignant tumor group, *n* = 32,539). The identified proportions of TB in the control and malignant groups are presented in Table 1. In total, 175 patients (1.35%) in the control group and 1776 patients (5.46%) in the study group had TB prior to the diagnosis of cancer (*p* < 0.0001). In 2010, the prevalence rate of pulmonary TB in the general population was 0.92-2.65% [19], which is comparable to the proportion of TB in the control group. In univariate analysis, TB was a risk factor for malignant cancer, in addition to traditional risk factors, such as age and sex (*p* < 0.0001). However, no significant difference in the proportion of TB was found between patients of Han and non-Han ethnicities.

### TB may be associated with an increased risk of specific cancer types

**Fig. 1:**
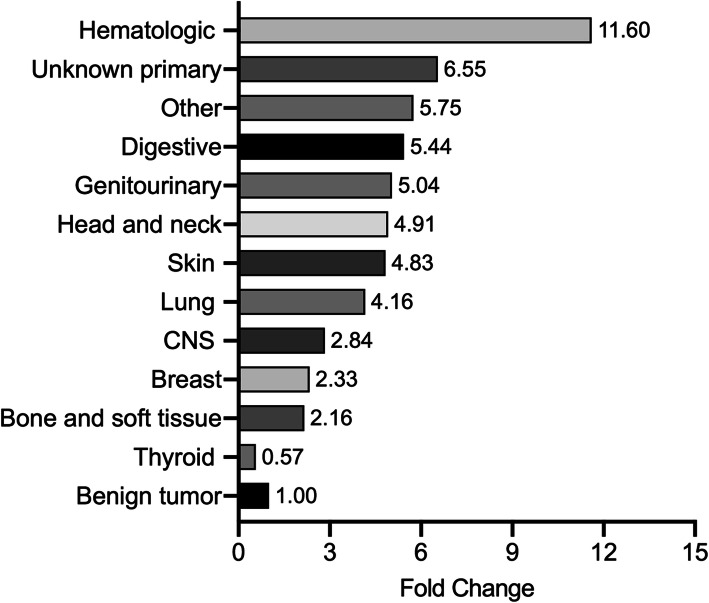
The fold change in the proportion of tuberculosis in patients with different cancer sites in the malignant group compared with the benign tumor group. Note, CNS, central nervous system

The proportion of TB may vary among patients with different cancer sites or types. The proportion of TB in patients with different cancer sites/types and the multivariate logistic regression analysis results stratified by cancer site/type and sex are shown in Table 2. The highest proportion of TB was found in patients with hematologic malignancies (15.66%), followed by those with unknown primary cancer (8.84%), “other” cancers (7.76%), digestive tract cancer (7.35%), genitourinary cancer (6.81%), head and neck cancer (6.63%), skin cancer (6.52%), central nervous system (CNS) cancer (3.83%), breast cancer (3.14%), and bone and soft tissue sarcoma (2.92%). In contrast, the lowest proportion of TB was found in patients with thyroid cancer (0.77%). The fold change in the proportion of TB for different cancer sites in the study group compared with the control (benign tumor) group is also shown in Fig. 1.

**Table 2:** Proportion of tuberculosis (TB) and cancer risk in patients stratified by tumor site

Cancer type (N.)	Total			Male			Female		
	N. of TB	%	Adjusted OR* (95% CI)	N. of TB	%	Adjusted OR^a^ (95% CI)	N. of TB	%	Adjusted OR^a^ (95% CI)
**Benign tumor (*n*** **= 12,916)**	175	1.35	Reference	26	3.27	Reference	149	1.23	Reference
**Malignant cancers (*n*** **=** 32,539**)**	1776	5.46	1.68 (1.43–1.97)	861	6.93	1.12 (0.79–1.60)	915	4.55	1.83 (1.53–2.19)
**Lung cancer** (*n* = 3968)	223	5.62	1.44 (1.06–1.95)	165	6.48	1.27 (0.83–1.94)	58	4.08	1.57 (1.04–2.37)
Adenocarcinoma (*n* = 2046)	91	4.45	2.05 (1.43–2.95)	59	5.58	1.72 (1.06–2.81)	32	3.34	2.09 (1.28–3.41)
Squamous cell carcinoma (*n* = 763)	57	7.47	1.35 (0.82–2.22)	50	7.37	1.53 (0.90–2.62)	7	8.24	NA
Small cell (*n* = 497)	25	5.03	NA	22	5.66	NA	3	2.78	NA
Other (*n* = 662)	50	7.55	1.38 (0.87–2.18)	34	8.04	1.44 (0.81–2.57)	16	6.69	NA
**Extrapulmonary tumor (*n*** **=** 28,571**)**	1553	5.44	1.72 (1.46–2.02)	696	7.05	1.17 (0.82–1.68)	857	4.59	1.88 (1.57–2.24)
**CNS (*n*** **=** 287**)**	11	3.83	NA	6	3.33	NA	5	4.67	NA
**Thyroid (*n*** **=** 4784**)**	37	0.77	0.30 (.021–0.44)	6	0.57	NA	31	0.83	0.42 (0.28–0.62)
**Head and neck** (*n* = 799)	53	6.63	1.52 (1.01–2.30)	35	6.52	1.09 (0.66–1.81)	18	6.87	NA
**Digestive (*n*** **=** 8722**)**	641	7.35	1.33 (1.05–1.68)	476	8.10	1.17 (0.80–1.70)	165	5.80	1.41 (1.05–1.88)
Esophagus (*n* = 1611)	241	14.96	2.23 (1.65–3.02)	185	16.53	2.49 (1.64–3.79)	56	11.38	1.91 (1.24–2.95)
Gastric-esophageal junction (*n* = 522)	46	8.81	0.80 (0.48–1.33)	38	8.70	0.69 (0.39–1.22)	8	9.41	NA
Stomach (*n* = 2055)	141	6.86	1.12 (0.80–1.56)	111	7.58	1.04 (0.68–1.60)	30	5.08	1.20 (0.76–1.90)
Colon, rectum and anus (*n* = 2424)	124	5.12	0.74 (0.53–1.03)	83	5.79	0.69 (0.44–1.08)	41	4.14	0.85 (0.55–1.29)
Liver (*n* = 1316)	53	4.03	0.50 (0.32–0.79)	41	4.05	0.53 (0.32–0.87)	12	3.96	NA
Biliary tract (*n* = 369)	19	5.15	NA	8	5.44	NA	11	4.95	NA
Pancreas (*n* = 425)	17	4.00	NA	10	3.82	NA	7	4.29	NA
**Hematologic malignancies (*n*** **=** 894**)**	140	15.66	4.88 (3.66–6.51)	88	18.14	4.07 (2.66–6.21)	52	12.71	4.87 (3.39–7.01)
Non-Hodgkin's lymphoma (*n* = 675)	113	16.74	4.57 (3.35–6.24)	71	19.72	4.27 (2.74–6.66)	42	13.33	4.21 (2.80–6.33)
Hodgkin's lymphoma (*n* = 122)	20	16.39	NA	12	18.46	NA	8	14.04	NA
Multiple myeloma (*n* = 87)	6	6.90	NA	4	7.41	NA	2	6.06	NA
Leukemia (*n* = 10)	1	10.00	NA	1	16.67	NA	0	0.00	NA
**Genitourinary (*n*** **=** 5711**)**	389	6.81	2.22 (1.82–2.70)	NA	NA	NA	NA	NA	NA
Cervix (*n* = 2839)	261	9.19	3.49 (2.79–4.38)	NA	NA	NA	NA	NA	NA
Uterus (*n* = 780)	22	2.82	NA	NA	NA	NA	NA	NA	NA
Ovary (*n* = 1045)	56	5.36	1.88 (1.33–2.64)	NA	NA	NA	NA	NA	NA
Prostate (*n* = 277)	16	5.78	NA	NA	NA	NA	NA	NA	NA
Bladder (*n* = 314)	17	5.41	NA	14	6.06	NA	3	3.61	NA
Kidney (*n* = 456)	17	3.73	NA	13	4.59	NA	4	2.31	NA
**Skin cancer (*n*** **=** 491**)**	32	6.52	0.84 (0.51–1.37)	15	6.05	NA	17	7.00	NA
Melanoma (*n* = 174)	12	6.90	NA	6	7.06	NA	6	6.74	NA
Nonmelanoma (*n* = 317)	20	6.31	NA	9	5.52	NA	11	7.14	NA
**Bone and soft tissue** (*n* = 718)	21	2.92	NA	9	2.59	NA	13	3.50	NA
**Breast (*n*** **=** 5443**)**	171	3.14	1.29 (1.02–1.62)	0	0.00	NA	171	3.16	1.36 (1.08–1.73)
**Unknown primary (*n*** **=** 181**)**	16	8.84	NA	8	8.16	NA	8	9.64	NA
Other* (*n* **=** 541**)**	42	7.76	1.93 (1.28–2.90)	10	4.46	NA	32	10.09	2.41 (1.53–3.80)

Notes: *NA* not applicable, *N* number

*, this category included cancers originating from the testis, vulva, penis, vagina, labia majora, labia minora, perineum, scrotum, hydatidiform mole, chorion, notochord, adrenal cortex, neuroendocrine system, mediastinum, sweat glands, mesothelium, thymus, umbilical tube and so on

OR, odds ratio; 95% CI, 95% confidence interval; CNS, central nervous system;

*The OR was adjusted by sex and age (as a continuous variable)

^a^ The OR was adjusted by age (as a continuous variable)

In the multivariate logistic regression analysis, TB remained an independent risk factor for all cancers (OR 1.68, 95% CI 1.43-1.97), including lung cancer (OR 1.44, 95% CI 1.0-1.95) and extrapulmonary cancer (OR 1.72, 95% CI 1.46-2.02), after adjusting for sex and age. Analysis stratified by specific types of cancer revealed high risks of non-Hodgkin’s lymphoma, cervical cancer, esophageal cancer, “other” cancer, ovarian cancer, head and neck cancer, and breast cancer and reduced risks of thyroid and liver cancer after TB. No statistically significant elevations in the risk of cancers originating from the gastric-esophageal junction, stomach, colon, rectum, anus, or skin were observed. With the exception of esophageal cancer and non-Hodgkin’s lymphoma, the relative cancer risk after TB was significantly elevated in females but not in males in the study group.

## Discussion

The current case-control study was performed in Xinjiang Province in China, where the prevalence of pulmonary TB is high [6, 20]. In this study, we found a greater proportion of patients with malignant cancers than of those with benign tumors who had a previous TB. Prior TB might increase the risks of lung cancer and some extrapulmonary cancers, including hematologic malignancies, esophageal cancer, genitourinary cancers, head and neck cancer, breast cancer, and “other” cancers. In accord with several prior studies [9, 11, 13, 15, 21], our results indicate an association between TB and certain cancer types (Table 3).

**Table 3:** A summary of studies of the risk of cancer in tuberculosis (TB) patients compared with the general population

	Type of TB infection	Type of study	Sample Size	Incidence of cancer in TB patients	Hazard ratio or relative risk	Extrapulmonary cancer type	PMID
Kuo et al. 2013	Latent	Retrospective population-based study	530	7.91%	All cancers: 2.07, 95% CI, 1.90-2.26 Nonpulmonary cancer: 1.71, 95% CI: 1.54-1.90	Head and neck, Digestive tract, Skin, Bladder, Hematological malignancies	23,652,313
Simonsen et al. 2014	Active	Cohort study	15,024	11.6%	All cancers: 1.52, 95% CI 1.45-1.59; Extrapulmonary cancer: 1.29, 95% CI: 1.22-1.36	Malignant pleural mesothelioma, Hodgkin’s lymphoma, ovarian, non-Hodgkin’s malignant lymphoma	25,216,835
Su et al. 2016	Latent	Retrospective population-based study	11,522	8.46%	All cancers: 2.29, 95% CI: 1.26-4.17 Nonpulmonary cancer: NA	Multiple myeloma, kidney, bladder, hepatobiliary, gastrointestinal	26,825,880
Everatt et al. 2017	Active	Retrospective cohort study	21,986	7.2%	All cancers: 1.76, 95% CI: 1.67-1.85; Nonpulmonary cancer: 1.41, 95% CI: 1.33-1.50	Mouth and pharynx, esophagus, stomach, larynx, cervix uteri, leukemia, hematological cancers, “other” cancers	28,572,839
Chen et al., 2020	Mixed	Case-control study	32,539 cases and 12,916 controls	NA	All cancers: 1.68, 95% CI: 1.43-1.97; Nonpulmonary cancer: 1.72, 95% CI: 1.46-2.02	Non-Hodgkin’s lymphoma, cervical cancer, esophageal cancer, “other” cancer, ovarian cancer, head and neck cancer, breast cancer	This study

*NA* not available

Chronic inflammation during TB may lead to cancer development [22-25]. However, the etiopathogenesis of this association is still imperfectly known. In lung cancer patients, TB-associated chronic inflammation in the lungs could cause clastogenic activity in the DNA of bronchial epithelium [22]. Furthermore, intracellular Mycobacterium TB DNA may induce neoplastic transformation of bronchial epithelial cells via lateral gene transfer [22]. Moreover, TB [26] and other chronic inflammatory disorders [27] might exert a huge influence on the secretory inflammation profiles of T helper cells and decrease antigen-specific T-cell responses in the pulmonary and lymphoid compartments [23].

Although far more males than females have TB globally [28, 29], TB-infected females than males often have more precarious social and economic positions [29, 30]. In this study, females seemed to have a higher risk of developing cancer than males after TB, although esophageal cancer and non-Hodgkin’s lymphoma were exceptions, suggesting that TB may contribute to the sex-specific development of malignant cancer. Indeed, estrogen signaling in females may contribute to a complex and sex-specific link between TB and cancer [31], such as TB could reprogram macrophages to a nonbactericidal M2 phenotype [28, 32-35]. In addition, some unadjusted confounding factors, such as autoimmune disorders, preferably occurred in females and were linked to lung and extrapulmonary cancer risk [36-38]. However, more future prospective studies on female sex and cancer risk are warranted.

Indeed, prior TB has been consistently found to be associated with a subsequent high risk of hematological malignancies, including non-Hodgkin’s lymphoma, Hodgkin’s lymphoma, multiple myeloma, and leukemia. In addition, more than one published study has shown that patients with TB are relatively more susceptible to cancer in the digestive tract, genitourinary tract, and head and neck. Consistent with these findings, we showed that TB patients have elevated risks of esophageal cancer, cervical cancer, head and neck cancer, and ovarian cancer. Furthermore, we showed that TB patients had an elevated risk of breast cancer. However, our data did not show that there was an elevated risk of skin cancer after TB. In contrast to the increased risk of hepatobiliary cancers shown by Su et al. [15], we found that TB has a protective effect against liver cancers (OR 0.50, 95% CI 0.32-0.79). However, the effect of TB on liver cancer is still controversial [39]. The differences in the risk of specific types of cancer risk in TB patients might be due to both differences in the study populations and differences in the research design.

This study was performed in a highly endemic area for TB with a large sample size. However, the current study was limited by estimating the real incidence of cancer after TB. In our study, we adjusted for age, sex, and ethnicity but lacked data to examine the role of tobacco smoking and other confounding factors associated with both TB and cancer. Furthermore, the misclassification of TB was also possible, although we included both active and inactive TB cases.

In clinical practice, clinicians should be alert to the association between cancer and TB. Some cohort studies have shown that the incidence of cancers in TB patients ranged from 7.2 to 11.6% (Table 3). The present study indicated that approximately 5% of cancer patients had prior TB in a highly endemic area in China. Indeed, some scientists have reported that the reactivation of latent TB infection has detrimental effects on outcomes in cancer patients during treatment with immune checkpoint inhibitors [40] and intensive chemotherapy [40-43]. Thus, we recommend screening for latent or active TB before initiating immunotherapy and intensive chemotherapy in most cancer patients. However, we were unable to calculate the benefits of this screening strategy in cancer patients. A future prospective study assessing the relationship of TB with prognosis in cancer patients is warranted.

## Conclusions

Our data showed that a greater proportion of cancer patients than benign tumor patients with a history of TB, indicating a high risk of cancer in TB patients. Prior TB may increase the risk of cancer, including hematologic malignancies, esophageal cancer, genitourinary cancers, head and neck cancer, breast cancer, and “other” cancers.

## Data Availability

The datasets generated and/or analysed during the current study are not publicly available due to privacy/ethical restrictions but are available from the corresponding author on reasonable request.

## Abbreviations

TB: Tuberculosis
OR: Odds ratio
CI: Confidence interval
CNS: Central nervous system
